# Supplementary material for: Two Novel SNPs in RET Gene Are Associated with Cattle Body Measurement Traits
Source: Animals (Basel). 2019 Oct 21;9(10):836. doi: 10.3390/ani9100836 (PMC6826558; doi:10.3390/ani9100836)
Supplement: Supplementary file 1 [file animals-09-00836-s001.pdf]

UUU 3.4(Phe 4)	UCU 11.9(Ser 14)	UAU 1.7(Tyr 2)	UGU 11.9(Cys 14)
UUC 5.1(Phe 6)	UCC 6.8(Ser 8)	UAC 3.4(Tyr 4)	UGC 11.1(Cys 13)
UUA 3.4(Leu 4)	UCA 0.9(Ser 1)	UAA 5.1(Stop 6)	UGA 20.4(Stop24)
UUG 1.7(Leu 2)	UCG 6.0(Ser 7)	UAG 3.4(Stop 4)	UGG 15.3(Trp 18)
CUU 31.5(Leu 37)	CCU 45.1(Pro 53)	CAU 17.9(His 21)	CGU 29.0(Arg 34)
CUC 30.7(Leu 36)	CCC 29.0(Pro 34)	CAC 23.0(His 27)	CGC 20.4(Arg 24)
CUA 16.2(Leu 19)	CCA 28.1(Pro 33)	CAA 35.8(Gln 42)	CGA 24.7(Arg 29)
CUG 29.0(Leu 34)	CCG 30.7(Pro 36)	CAG 29.8(Gln 35)	CGG 21.3(Arg 25)
AUU 3.4(Ile 4)	ACU 3.4(Thr 4)	AAU 0.9(Asn 1)	AGU 11.1(Ser 13)
AUC 2.6(Ile 3)	ACC 4.3(Thr 5)	AAC 4.3(Asn 5)	AGC 11.1(Ser 13)
AUA 1.7(Ile 2)	ACA 2.6(Thr 3)	AAA 8.5(Lys 10)	AGA 14.5(Arg 17)
AUG 1.7(Met 2)	ACG 3.4(Thr 4)	AAG 2.6(Lys 3)	AGG 7.7(Arg 9)
GUU 9.4(Val 11)	GCU 40.0(Ala 47)	GAU 22.1(Asp 26)	GGU 22.1(Gly 26)
GUC 13.6(Val 16)	GCC 31.5(Ala 37)	GAC 10.2(Asp 12)	GGC 28.1(Gly 33)
GUA 11.9(Val 14)	GCA 18.7(Ala 22)	GAA 24.7(Glu 29)	GGA 56.2(Gly 66)
GUG 11.9(Val 14)	GCG 19.6(Ala 23)	GAG 17.9(Glu 21)	GGG 24.7(Gly 29)

Supplementary Figure S1 The distribution of genetic codons of bovine *RET* gene.

Note: The underlined bases represent synonymous mutations.

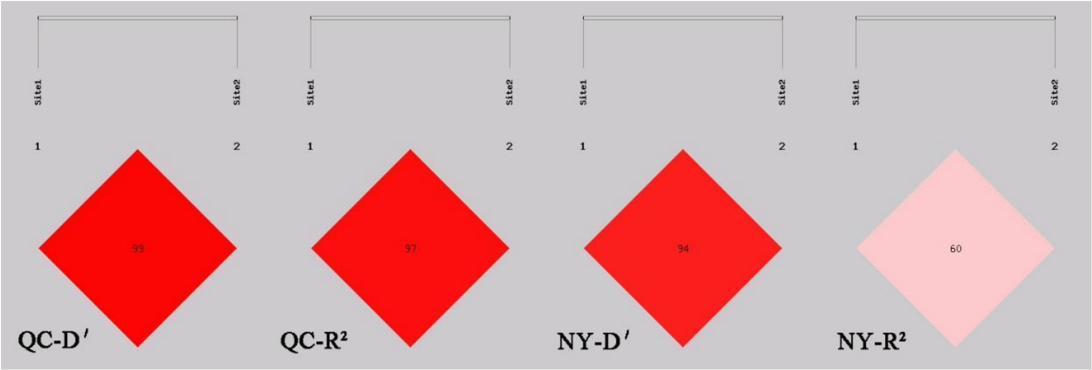

Supplementary Figure S2 Linkage disequilibrium plot of two SNPs in bovine *RET* gene.

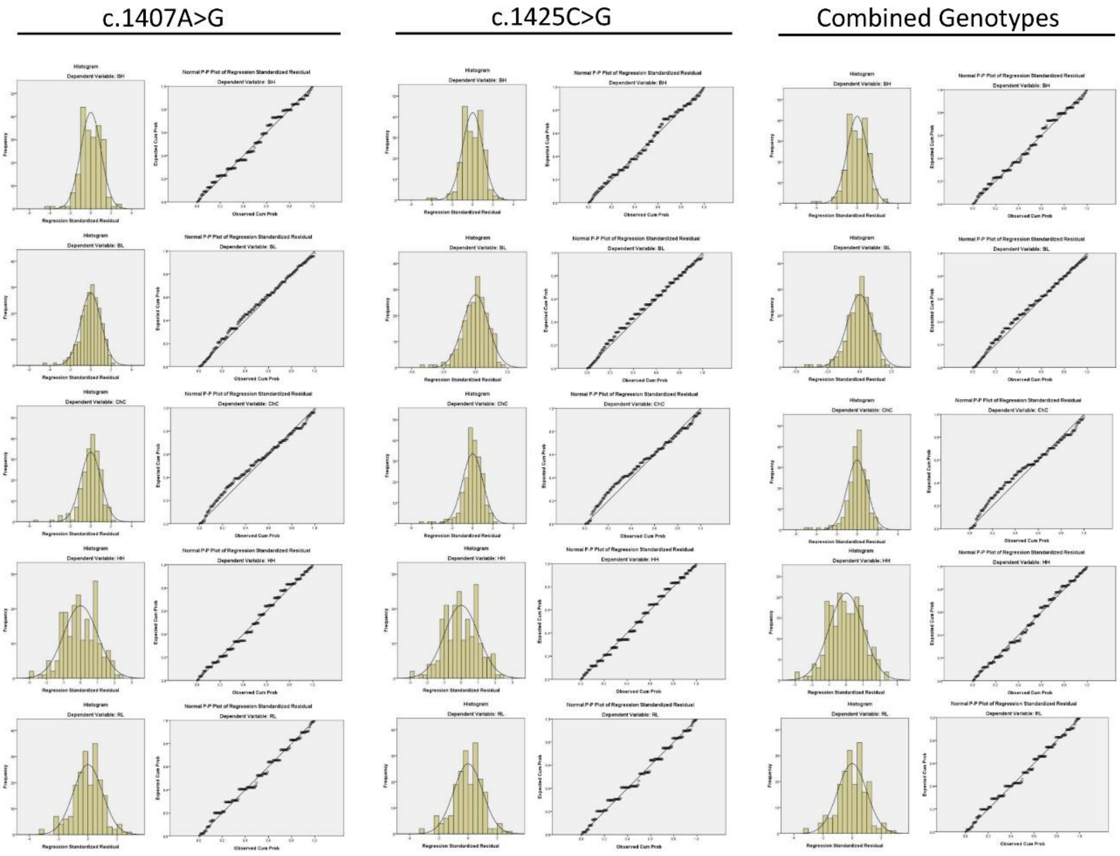

Supplementary Figure S3 Graphical tests to explore normality of the residual in Qinchuan group. The left row of each SNP is the histogram and the right row is the normal P-P plot of regression standardized residual. Different line was shown the residual of the genotypes and different body measurement.

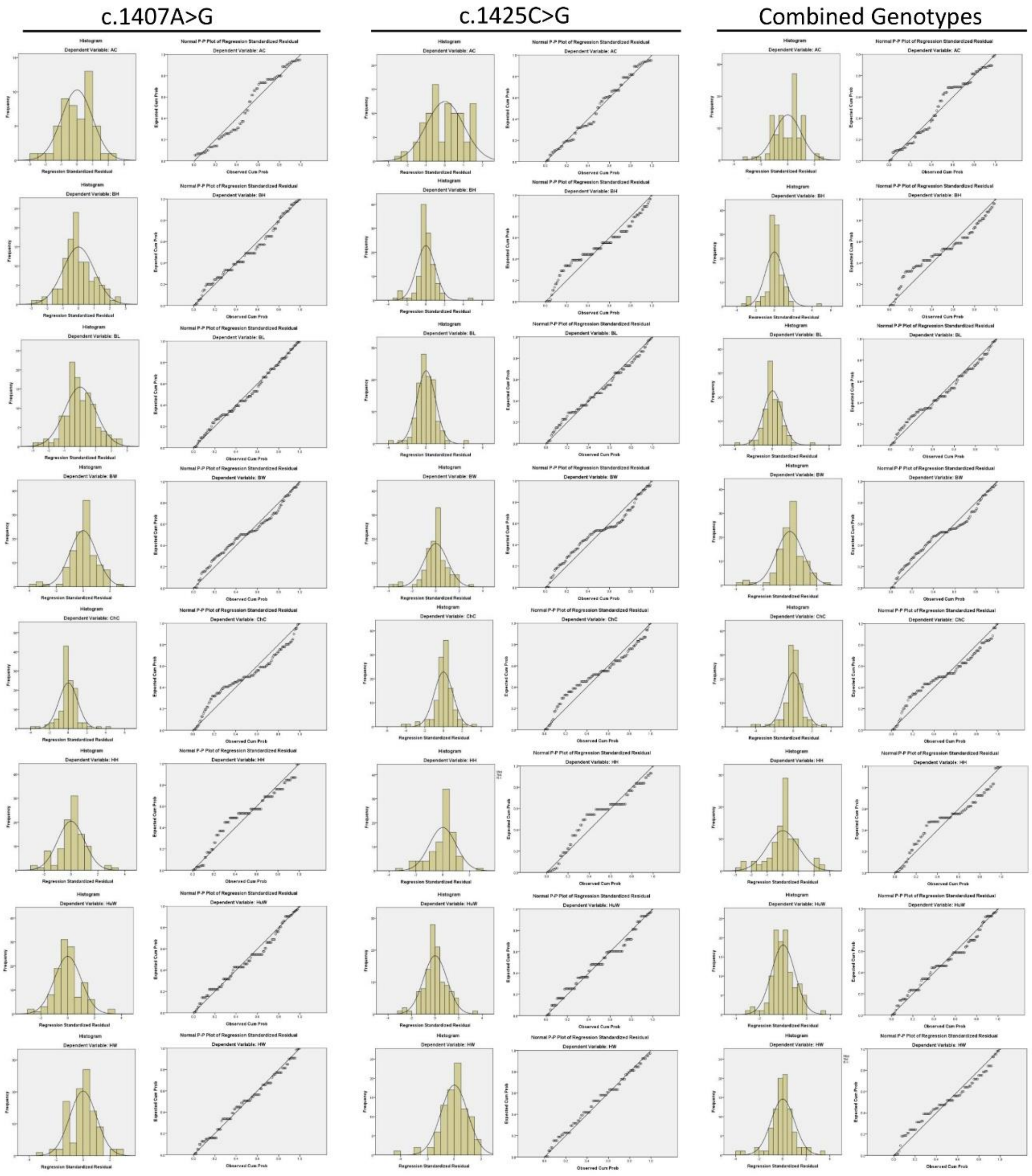

Supplementary Figure S4 Graphical tests to explore normality of the residual in Nanyang group. The left row of each SNP is the histogram and the right row is the normal P-P plot of regression standardized residual. Different line was shown the residual of the genotypes and different body measurement.
